# Reflex Neurosis in Relation to Dental Pathology

**Published:** 1889-11

**Authors:** George B. Hayes


THE
AMERICAN JOURNAL
—OF—
DENTAL SCIENCE.

VOL. XXIII. BALTIMORE, NOVEMBER, 1889. NO. 7.

ARTICLE I.

### REFLEX NEUROSIS IN RELATION TO DENTAL PATHOLOGY.

BY GEORGE B. HAYES, D. D. S.

Among the various works on medicine and surgery in clinical reports and especially in dental literature are to be found cases of nervous derangement in which portions of the nervous system have been affected by dental irritation or have been implicated in the inflammatory results of tooth disease. Such cases widely diverse in character and in the extent to which the normal functions are impaired form at once an interesting subject for study, and illustrate how serious may be some of the maladies dependent on tooth disease, also how largely the pathology of the teeth is associated with serious morbid changes in contiguous structures.

Under the head of such affections may be observed two classes of reflexes: first, those of dental origin; second, those whose manifestations appear in the teeth but are caused by distant irritation. A pain in a tooth is not indicative of the source of trouble, as repeated attempts at treatment have often demonstrated. The cause may be remote or in another tooth. Again, without the slightest subjective intimation as to their origin, abnormal conditions of the teeth may induce most obstinate disorders of the head, body, or extremities; or, as it has been tersely put, "one may have toothache in the brain, stomach, and arm or, headache, gastralgia, etc., in the teeth."

The normal reflex, by which an afferent current from an irritated point to a central ganglion is returned as a centrifugal impulse to excite activity in one or more of the normal functions is well understood and need not take our attention, but it is the derangement of this healthy activity in its unaccountable manifestations, that at once interests and puzzles us. Any attempt at accurately tracing the misconducted routes of such impulses, or the reasons of such deroutation is confronted by many obstacles and in the present state of neurology must be purely theoretical and highly unsatisfactory.

To help our understanding of the intricacy of the nervous system it has been compared to the "intricate switch board of a huge telegraph office where are focalized, myriads of wires from all parts of the compass. When in normal action, the connections are such that a message from any peripheral point is shunted to its proper receiver, transferred to another wire, or sent into other and higher offices. Subordinate ganglia and spinal centers may be looked upon as relay or local stations with limited authority and power, but which in extreme or important cases must refer to a more central authority. As far as concerns any adequate comprehensions of the mysterious workings of the medulla, either in health or disease, we are very much like an individual wholly ignorant of and standing before the switchboard and unheard of telegraph machines." It is to the disordered states of this complicated system that our attention is directed, and for convenience of classification we can do little better than follow the summary as given by Dr. Brubaker in the American System of Dentistry. Under the first great division of reflex odontalgia may be noted cases whose origin is: 1st, *Peripheral*, including dental, nasal, ocular, and visceral; 2nd, *Cerebral*, including thrombi inflammation, tumors, and hysteria; 3rd, *Systemic*, including malaria, gout, syphilis and constitutional derangements. The first of these cases of odontalgia, in which the sensation of pain is located in another tooth on the same or opposite side to the seat of the trouble, is undoubtedly the most common that comes under the dentist's observation and is so misleading that serious impairment is liable to follow mistaken diagnosis. The physiological or pathological course of the reflex, ascribed by many to sympathe ic action, is the most satisfactory explanation. A single illustration may be taken as typical although the greatest diversity may obtain in details. Friedberg, in Virchow's Archives, makes allusion to a case of severe suffering for twelve days by a patient who begged to have the second and third molars extracted. Both were sound but the central incisor was carious, though indolent to percussion. This was extracted and the odontalgia at once ceased.

In cases of nasal and ocular origin, and the following heads most careful diagnosis must be taken, inasmuch as such cases are usually presented to the dentist under mistaken ideas as to the origin of the trouble. Catheterization of the nasal ducts, ocular injections and inflammation of the eyes are aggressive causes.

The field embraced under the head of cases of visceral origin is large and includes irritations of the alimentary tract, dyspepsia, sea-sickness, nausea, hunger, morbid conditions of the uterus, in both pregnant and non-pregnant states. Many cases have been reported by gynecologists during pregnancy as the whole system, and especially the teeth, are liable to suffer from lack of nutrition during gestation.

Dr. Storer relates the case of a young woman of good health and physique, four months and a half gone with her first child: "Her general health had been good and until the present had never suffered from a neuralgic pain; reported excessive tooth-ache of nearly two months standing. It commenced on the left side of the lower maxilla and then affected both sides of the jaw; that during the whole period she had been under a physician's charge, had been thoroughly treated with anodynes, local and general, by antiperiodics, purgatives, fomentations, and counteriritants; that a tooth, the only carious one she had, had been extracted ten days previous; and that it had been proposed to remove others, to which she would not consent. All without relief. She was ordered a fragment of pellitory root, pyrethrum, as a direct gingival stimulant and on the second day presented herself cured, without return of the malady." Such cases, harrassing as they may be, often render extraction and radical treatment questionable as such has frequently caused abortion.

Cerebral diseases, for example, insanity, softening of the brain, tumors and inflammation may produce odontalgia, but clinical reports reveal comparatively few, inasmuch as owing to their obscurity positive diagnosis is often rendered difficult. Dr. Benson relates a case of a young woman who suffered from severe pain due, as was subsequently shown, to be from an abscess at the origin of the fifth nerve.

In very many malarial districts, even in Michigan, tooth-aches of a distinctly periodic character are observed which are only relieved by antiperiodics, quinine, arsenic, etc. Many other systemic diseases as rheumatism, gout and specific diseases like syphilis, affect the dental organs with severe and obstinate odontalgia. The painful and serious results of over-medication with mercury and the iodides belong in this category, as due to systemic derangement.

Having briefly passed in review the causes that falsely register pain in the teeth there remains for consideration the second great division of our subject, namely, those disturbances, peripheral, nervous and cerebral—distant and neighboring—that may be set up by pathological conditions of the teeth as primary inciting causes. Under this head ocular and aural troubles predominate, owing probably to situation and nervous relations. Cases are on record illustrating abnormal conditions of almost every part of the eye, with paresis of the recti, palpebræ and ciliary muscles. The following case, singular in its manifestations, exemplifies the irregular and unpredicable courses of many abnormal reflexes.

A young girl, æt. fifteen years, subject to copious lachrymation on each occasion that she left the house for out door exercise. There was no outward manifestation of organic disease, the eyes looking brilliant and healthy; still the moment she went into the open air tears poured down her cheeks in the most distressing manner. After several months suffering she was brought under a dentist's notice to see if the teeth could in any way influence the condition of affairs. On examination the cuspidati of the upper jaw were absent though the dental arch was perfect. Understanding that no teeth had been extracted from the child, the first bicuspids were removed. Within a week manifest improvement were observable and within three months the cuspidati made their appearance.

As illustrative of the frequency of aural troubles Sexton, in reviewing the records of 1,500 cases, says that "perhaps one-third owe their origin or continuance in a greater or less degree to diseases of the teeth." An interesting case of deafness cured by extraction of a tooth is recorded by Dr. Campbell: Mrs. A., æt. thirty-eight years, presented herself to have several teeth extracted. Upon removal of a cuspid tooth the patient remarked that she could now hear with her left ear. She had been entirely destitute of hearing in that ear for upwards of three years, having lost the faculty while attending church. She said that immediately after the removal of the cuspid tooth a sensation followed like the passage of a current of air in the ear; immediately afterward she recovered her hearing which was permanent.

Muscular disorders from another class of cases under this heading in which active contraction or spasm is brought about by dental irritation. Chief among these are the muscles applied by the facial nerve, the motor branch of the geminus, and the external branch of the spinal accessory. Facial spasm often called "painless facialtic" may occur in the tonic or chronic form, the spasms varying from a few moment to hours. Trismus, or contraction of the muscles of mastication, is a common result of the impaction and irregular eruption of the wisdom teeth. Over crowding of the teeth, as in the following case, is a fair example of many similar ones reported in medical and dental jouanals: "A gentleman, æt. thirty years, had been suffering from lock-jaw and pain under the right ear for the past twelve months; could separate the jaws in front only about half an inch. He attributed the mischief to a cold and had been subjected to various kinds of treatment, including leeches, blistering, etc., without benefit. The teeth were observed to be much crowded and wedged, particularly in the upper jaw, which was concluded to be the causc of suffering; accordingly one of the anterior molar teeth was extracted, The tooth was large but perfectly sound, and the patient returned home in a couple of weeks cured."

Turning to clinical medicine for records of visceral disorders due to reflected irritation from the teeth, we are disappointed in not finding more specific cases. However, the following are a few of those recorded, details of which space alone prevents: "Obstinate vomiting resulting in weakness and inability to retain food, due to deposits of salivary calculus." "Vomiting, convulsions and death from painful dentition." "Uterine neuralgia from eruption of wisdom teeth."

Facial and other neuralgias are widely recognized not only as common, but often as serious complications of dental troubles. Of these the most manifest are those coming from the fifth pair of nerves on account of their anatomical association. In general a tendency to facial pain is an accompanying symptom of impaction and alveo-dental abscess. Neucourt went so far as to lay down a serious of rules by which to diagnose such neuralgia from that of other causes than dental, and cited seventeen cases of neuralgia illustrating his rules. It may not be amiss to briefly mention these rules as their general application will often prove valuable. They are given in the *American System of Dentistry*, as follows:

1. "When a tooth is in itself the seat of pain and the patient definitely specifies it as such, there can be no doubt that it is the origin of the trouble.

2. When in addition to the pain there follows a swelling of the cheek and gum, resulting in abscess, the indication is specific; if a tooth is sensitive to percussion, seems longer than others, the indication is decisive whether it is carious or not.

3. Even if the pain is diffused and spread over the side of the face, and attended with distinct exacerbations, thus presenting all the signs of typical facial neuralgia, nevertheless, if it eventually localizes and limits itself in the region of the dental arch, accompanied with pain, redness, and swelling with extreme sensibility to pressure, possibly terminating in an abscess, the disturbance is, in such cases, of dental origin.

4. Another point serving to distinguish neuralgia of dental origin from that due to other causes is the continual agitation and discomfort of the patient. There are no such periods of calm as occur in neuralgia from other causes. The pulse is accelerated and hard, and there is at times general sweating."

A case or two will suffice as examples. First, facial neuralgia of one year's duration cured by the extraction of a carious tooth: "A man, æt. thirty-two years old, in good health; first experienced pain about the head in consequence of a severe cold. These, under house treatment, were succeeded by pains about the cheeks, sometimes in the upper, again in the lower jaw, and upon either side of the face. There was then a space of a few weeks of relief, when lancinating pains were again felt in the right cheek shooting about the face from eyes to jaw; then periods of relief and returning attacks followed each other, the eyes, forehead, and side of the head gradually becoming implicated; in severe assault the eyelids, lips and even the muscles of the face were seized with spasmodic twitchings. Toothache, properly speaking, he had not, the pains often shooting toward, rather than from the teeth. The patient himself had had two teeth extracted in hope of relief, but in vain. After about eleven months of continuous suffering, upon advice of a surgeon, another tooth was extracted though it only showed a slight carious imperfection, and the patient had never suspected any trouble from it. It proved to have attached to its roots a fungoid growth. The pain at once stopped without return."

The second case is "neuralgia of the arm from carious teeth and undue pressure of artificial teeth related by Mr. Salter, of a lady under his care who had lost all her teeth and was then wearing an artificial set. One curious fact was constantly observed in the progress of her case. When any one of the teeth in the lower jaw became irritable or tender from caries, she was immediately attacked with severe neuralgic pain at a spot, small and circumscribed on the front of the left-arm about two inches below the line of flexion of the elbow joint; and what is more remarkable is that when the artificial teeth hurt the lower jaw on that side the same symptom manifested itself. The right side had never been similarly affected."

Neuralgia of the face, neck and arm, with paralysis of the latter, from carious wisdom teeth, sciatics, neuralgia from fragment of root in alveolus, from osteo-dentine, exostosis, and ulceration of the gums are among many other similar cases.

There is another category of affections that may be considered as secondary and more remote including disturbances of the cerebral centers, *e. g.*, epilepsy, hysteria, headache and insanity. The irregularity and intensity of such reflexes is widely varied. The susceptibility of some persons may be noted as often amounting to an idiosyncracy. In persons of such a nervous or neuralgic diathesis, whether congenital or acquired, the slightest attack of exhaustion or depression may set up derangements resulting seriously. Epilepsy, whether hereditary as many writers claim or induced, has been known to be excited by dental troubles and cured by the removal of the cause, as the following case will testify: "A lad, a farmerboy, from Windsor, was admitted to the hospital for epilepsy. The usual remedies were tried for six weeks without effect. His mouth was then examined and the molar teeth of the lower jaw found to be much decayed, and of some of these only the roots remained. He did not complain of pain in the diseased teeth or in the jaw. The decayed teeth were, however, removed and the roots of each found to be enlarged from exostosis. During the eighteen months that followed he had not suffered from a single fit though for many weeks previous he had two or three per day." The constitutional condition known as hysteria, also considered an hereditary disease, may be induced by sickness, insufficient nourishment, diseases of the uterus, etc., and when the predisposition is established may be excited by almost any peripheral disturbance. Primary among them are morbid conditions of the teeth. Under the head of insanity, Esquiral says, "Among subjects of a lymphatic and nervous temperament the pains of first dentition sometimes become the cause of insanity. The appearance of the teeth through the gums causes all the symptoms to cease. I have observed this in the case of three young girls—they had convulsions, bloating of the face and discharge of saliva. I could not be deceived as to the cause of this malady—the delirium ceased at the end of a month and two teeth burst their envelopes. Fifteen days after the mania reappeared, with the same intensity; the gums of the late tooth were swollen and red. The attack lasted several months and only ceased on appearance of the teeth."

This concludes a very meager consideration of many of the principal reflexes associated with dental pathology. Many others may need only a little more careful observation and refined diagnosis to be embraced under the same head. Although much has been written concerning reflex neurosis and the theories of their derangement, the present state of neurology offers no satisfactory explanation of the causes, nor attempts to prophesy as to the course of such misdirected currents. The questions still remain puzzling but profound in the interest of many a patient sufferer. Such a review of cases with a careful study of the subject can not fail in its impression of the importance of healthy dental organs and the intimate relations they sustain in their abnormal conditions to the whole body. From the infant, fretting under the first pains of dentition, to the neuralgia of adentulous old age, there is no stage that is exempt from the harrassing results of a deranged nervous system, either affecting the teeth or affected by them. No subject can better illustrate the overlapping and interdependence of the sciences of medicine and dentistry. The physician, the ophthalmologist, the aurist must recognize each his intimate association with the dental surgeon in the great healing art. From all this the practical conclusion is manifest. The successful dentist of to-day can no longer limit his powers by his fingers; the times demand a wider knowledge and higher training to guard against errors in diagnosis and treatment heretofore insufficiently recognized.
—*Dental Register.*

